# Dietary and genetic risk scores and incidence of type 2 diabetes

**DOI:** 10.1186/s12263-018-0599-1

**Published:** 2018-05-16

**Authors:** Ulrika Ericson, George Hindy, Isabel Drake, Christina-Alexandra Schulz, Louise Brunkwall, Sophie Hellstrand, Peter Almgren, Marju Orho-Melander

**Affiliations:** 1 Diabetes and Cardiovascular Disease, Genetic Epidemiology, Department of Clinical Sciences, Malmö, Lund University, Malmö, Sweden; 2 Clinical Research Centre, Building 60, floor 13, SUS in Malmö, entrance 72, Jan Waldenströms gata 35, SE-205 02 Malmö, Sweden

**Keywords:** Diet, Food intake, Gene-environment interactions, Cohort study, Type 2 diabetes

## Abstract

**Background:**

Both lifestyle and genetic predisposition determine the development of type 2 diabetes (T2D), and studies have indicated interactions between specific dietary components and individual genetic variants. However, it is unclear whether the importance of overall dietary habits, including T2D-related food intakes, differs depending on genetic predisposition to T2D. We examined interaction between a genetic risk score for T2D, constructed from 48 single nucleotide polymorphisms identified in genome-wide association studies, and a diet risk score of four foods consistently associated with T2D in epidemiological studies (processed meat, sugar-sweetened beverages, whole grain and coffee). In total, 25,069 individuals aged 45–74 years with genotype information and without prevalent diabetes from the Malmö Diet and Cancer cohort (1991–1996) were included. Diet data were collected with a modified diet history method.

**Results:**

During 17-year follow-up, 3588 incident T2D cases were identified. Both the diet risk score (HR in the highest risk category 1.40; 95% CI 1.26, 1.58; *P* trend = 6 × 10^−10^) and the genetic risk score (HR in the highest tertile of the genetic risk score 1.67; 95% CI 1.54, 1.81; *P* trend = 7 × 10^−35^) were associated with increased incidence of T2D. No significant interaction between the genetic risk score and the diet risk score (*P* = 0.83) or its food components was observed. The highest risk was seen among the 6% of the individuals with both high genetic and dietary risk scores (HR 2.49; 95% CI 2.06, 3.01).

**Conclusions:**

The findings thus show that both genetic heredity and dietary habits previously associated with T2D add to the risk of T2D, but they seem to act in an independent fashion, with the consequence that all individuals, whether at high or low genetic risk, would benefit from favourable food choices.

**Electronic supplementary material:**

The online version of this article (10.1186/s12263-018-0599-1) contains supplementary material, which is available to authorized users.

## Background

The prevalence of type 2 diabetes (T2D) is increasing worldwide, and it is of great concern to identify modifiable lifestyle factors including diet. However, both lifestyle and genetic predisposition determine the development of the disease [1], and some studies have indicated interactions between specific dietary components and individual genetic variants [2, 3]. Yet, few findings have been replicated, and it is unclear whether the importance of overall dietary habits, including T2D-related food intakes, differs depending on overall genetic predisposition to T2D.

Single nucleotide polymorphisms (SNPs) associated with T2D have been identified in genome-wide association studies (GWAS) [4, 5]. Moreover, high intakes of processed meat [6–8] and sugar-sweetened beverages (SSB) [9–11] have in meta-analyses of observational studies consistently been associated with increased risk of developing T2D, whereas high intakes of whole grain [12, 13] foods and coffee [14, 15] have been associated with decreased risk. Probable mechanisms behind these associations have been proposed [16–19]. Inverse associations with intakes of fruits and vegetables [20, 21], dairy products [22] (especially fermented dairy) [22–24]) and fatty fish [25] have also been observed, but these findings are less conclusive [23, 26, 27] or may be explained by intake of specific products within these food groups [20, 21, 23, 24, 28–30].

It has been indicated that associations between western dietary patterns and T2D may differ between individuals depending on genetic susceptibility, but dietary habits assessed by a Mediterranean diet score were not found to interact with a genetic risk score (GRS) for T2D in the EPIC InterAct study [31]. However, we are not aware of any previous study examining whether a diet risk score (DRS), based on specific food intakes previously found to associate with T2D, interacts with a GRS. Besides, a diet quality index, based on Swedish nutrition recommendations, that has been associated with cardiovascular disease could not be linked to incidence of T2D in the Malmö Diet and Cancer (MDC) Study [32], suggesting that the index components chosen to reflect overall diet quality may not capture food intakes of particular relevance in the development of T2D.

Our aim was to examine T2D incidence in the MDC study according to a DRS of the four foods and beverages most consistently reported to associate with T2D in epidemiological studies (processed meat, sugar-sweetened beverages, whole grain and coffee), and a GRS of 48 GWAS-identified T2D SNPs [5], as well as their interaction. We also examined interactions between the GRS and each of the diet components included in the DRS.

## Methods

### Study population and data collection

The MDC study is a population-based prospective cohort study in southern Sweden with baseline examinations in 1991–1996. Women born in 1923–1950 and men born in 1923–1945, living in the city of Malmö, were invited. Details of the cohort and the recruitment are described elsewhere [33]. The participants filled out questionnaires covering socio-economic and lifestyle and underwent a diet history assessment. Anthropometric measurements were conducted by nurses. Body composition was estimated with a bioelectrical impedance analyser. During the screening period, 28,098 participants (40% of the eligible persons) completed all baseline examinations. Of the non-participants, 49% did not reply to the invitation letter, 39% answered that they were not willing to take part, 7% died or moved before they had received an invitation and 5% failed to complete all baseline examinations.

We excluded 1230 participants, based on self-reported diabetes diagnosis, self-reported diabetes medication or information from medical registries (see below). We were then left with 26,868 individuals, of whom 25,430 individuals had available DNA, and out of those, 25,069 individuals were successfully genotyped for > 60% of the SNPs included in the GRS; these individuals constituted our study population. A random 50% subsample of those who participated in MDC study between 1991 and 1994 were invited to be involved in additional baseline examinations. All additional measurements were made at baseline with a median time lag of 7 months after the first visit. In total, 6103 individuals participated in the additional examinations (the MDC cardiovascular sub-cohort, MDCS-CC). Out of those, 4193 individuals were successfully genotyped for additional SNPs included in an extended GRS. The ethical committee at Lund University has approved the study (LU 51-90), and the participants have given their written informed consent.

### GRS for T2D

Weighted GRSs for T2D was calculated in PLINK from 48 T2D SNPs identified in 37 GWAS and confirmed or identified in a meta-analysis by Morris et al. [5] (Additional file 1: Table S1). Out of 63 identified SNPs in Morris et al., 9 were excluded as they were in linkage disequilibrium with other SNPs that we included in the GRS (rs6795735, rs10440833, rs1920792, rs4430796, rs757110, rs1387153, rs13081389, rs12255372 and rs4760790), 2 were excluded due to sex-specific associations in GWAS (rs11063069 and rs8108269) [5], 1 due to imprinting (rs2334499) [34] and 3 due to deviation from HWE (rs757210, rs4607517 and rs2796441) in our study. If the individuals had missing values for any of the 48 SNPs, it was substituted in PLINK by the mean of the risk alleles for that SNP calculated from the other individuals. Genotypes at each locus were coded as 0, 1 and 2, according to the number of T2D-increasing risk alleles, and a weighted GRS was calculated in PLINK such that each risk allele was weighted by their previously published effect sizes [5]. The weighted GRS was divided into tertiles.

### Extended GRS for T2D for secondary analyses in a subsample

An extended weighted GRS for T2D was calculated in PLINK from 68 T2D SNPs in a subsample of the MDC cohort (individuals with genotype information on 68 T2D SNPs and without prevalent diabetes from the MDC cardiovascular sub-cohort). The extended GRS included 20 of the additional SNPs identified after the meta-analysis by Morris et al. until Fuchsberger et al. [35] (Additional file 1: Table S9).

### Genotyping

A MALDI-TOF mass spectrometer (Sequenom MassArray, Sequenom, San Diego, CA) was used to genotype DNA samples using Sequenom reagents and protocols. Proxy SNPs were identified using SNAP version 2.2.2 when commercial primers were not available. SNPs that failed Sequenom genotyping were genotyped individually using TaqMan or KASPar allelic discrimination on an ABI 7900HT (Applied Biosystems, Life Technologies, Carlsbad, CA). All included 48 SNPs had genotyping success rate above 93%, and 45 of the SNPs had a success rate above 95%. The concordance rate was > 99% for all 48 SNPs, including the three SNPs with success rates between 93 and 95%, in 5500 samples which were additionally re-genotyped using Human Omni Express Exome Bead Chip Kit (Illumina, San Diego, CA, USA).

### Dietary data

Dietary data was collected once during the baseline period. An interview-based, modified diet history method was used that combined (i) a 7-day menu book for recording of intakes from meals that vary from day to day (usually lunch and dinner meals) and cold beverages, (ii) a 168-item diet questionnaire for assessment of consumption frequencies and portion sizes of regularly eaten foods not covered by the menu book and (iii) a 45-min interview. The MDC method has previously been described in detail [36].

Diet analyses were adjusted for the variable “diet method version”, because slightly altered coding routines of dietary data were introduced in September 1994 to shorten the interview time (from 60 to 45 min). This resulted in two slightly different method versions without major influence on the ranking of individuals [36]. The relative validity of the original MDC method was evaluated in the Malmö Food study 1984–1985, comparing the method with 18-day weighed food records [37, 38]. The Pearson correlation coefficients, adjusted for total energy, were in women and men respectively for fibre 0.69/0.74, bread 0.58/0.50, cereals 0.73/0.74, meat 0.92/0.84, fruits 0.77/0.60, vegetables 0.53/0.65, milk 0.84/0.83, cheese 0.59/0.47 and fish 0.70/0.35 [37, 38].

Food intakes were converted to nutrient intakes using the MDC nutrient database where information comes from the Swedish National Food Agency. Portions (instead of grams) were used in order to analyse the sum of whole grain products and the sum of fermented dairy products, because water contents and portion sizes differ. Standard portion sizes from the MDC study or from the National Food Agency were used [39]: fibre-rich soft bread (50 g/portion), fibre-rich crispbread (30 g/portion), breakfast cereals (25 g/portion), yoghurt (200 g/portion) and cheese (20 g/portion).

Food variables were natural logarithm transformed to normalize the distribution. To handle log transformation of zero intakes, we added 0.01. Energy-adjusted variables were obtained by regressing the variables on non-alcohol energy intake. Tertiles were used as exposure categories. As more than 33% were zero-consumers of SSB, these individuals constituted the lowest intake category and the higher categories were defined as below or above the median among the consumers.

### DRS for T2D

A DRS for T2D was constructed by classifying the individuals according to low, medium and high intakes of foods previously shown to consistently associate with incident T2D in meta-analyses of prospective cohort studies, as described in the introduction, i.e. processed meat (sausage and cured meat), SBB (beverages sweetened with energy containing sweeteners; mainly sucrose), whole grains (fibre-rich breads and cereals) and coffee (total; very few consumed decaffeinated coffee). High points were assigned for intakes expected to associate with increased T2D risk based on the earlier studies. Unweighted diet risk levels were used, as different diet assessment methods and intake levels in published studies complicate extrapolation to absolute risk estimates. Thus, for processed meat and SSB, no points were assigned to those with low intake, 1 to those with medium intake and 2 points to those with high intake. For whole grains and coffee, no points were assigned to those with high intake, 1 to those with medium intake and 2 points to those with low intake. Finally, the points were summed up to the risk score that was divided into three groups: low DRS (0–2 points), medium DRS (3–5 points) and high DRS (6–8 points).

### Extended DRS for T2D for secondary analyses

Although the most consistent associations with T2D have been observed for the food components we included in our DRS, some studies have also indicated that intake of other foods may be associated with risk of T2D. A recent meta-analysis suggested high intake of fruit to be protective [20], but the latest meta-analysis indicated no additional risk decrease at intakes above two servings per day [26]. Likewise, a non-linear association was seen for vegetable intake [26], although high intakes of specific types of vegetables, especially green leafy vegetables may be beneficial [20, 21, 28, 40]. Moreover, total intake of fruits and vegetables does not seem to associate with the risk of T2D [20, 26, 28, 40]. Dairy products have been suggested to be protective [22], especially fermented dairy products [23] such as yoghurt and cheese [22, 24], but it is unclear whether specific dairy foods or dairy components explain observed associations [23, 29, 30]. Lastly, findings regarding fatty fish are non-conclusive [25, 27].

For secondary analyses, extended scores were created that additionally included intakes (in tertiles) of fruit and vegetables, fermented dairy or high-fat fish. Thus, each extended score is included in total five foods or beverages. The extended scores summed up to low (0–3 points), medium (4–6 points) and high (7–10 points) DRS. Finally, we constructed an extended risk score simultaneously including intakes of the original components and all three additional components: low (0–4 points), medium (5–9 points) and high (10–14 points) DRS.

### Ascertainment of T2D incidence

We identified 3588 incident cases of T2D during 433,888 person-years of follow-up via at least one of seven registries (90% of cases) or at examinations during follow-up (10% of cases). The mean follow-up time was 17 ± 5.6 years (range 0–24). The subjects contributed person-time from date of enrolment to date of diabetes diagnosis, death, migration from Sweden or end of follow-up (December 2014), whichever occurred first. During follow-up, 0.5% had migrated from Sweden. If available, we used information on date of diagnosis from two registries prioritized in the following order: (i) the regional Diabetes 2000 registry of Scania [41] and (ii) the Swedish National Diabetes Registry [42]. These registries required a physician diagnosis according to established diagnosis criteria (fasting plasma glucose concentration ≥ 7.0 mmol/L or fasting whole blood concentration ≥ 6.1 mmol/L, measured at two occasions). Individuals with at least two HbA1c values above 6.0% with the Swedish Mono-S standardization system (corresponding to 6.9% in the US National Glycohemoglobin Standardization Program and 52 mmol/mol with the International Federation of Clinical Chemistry and Laboratory Medicine (IFCC) units) [43, 44] were categorized as diabetes cases in the Malmö HbA1c Registry. In addition, cases were identified via registries from the National Board of Health and Welfare: the Swedish National Inpatient Registry, the Swedish Hospital-based outpatient care, the Cause-of-death Registry and the Swedish Prescribed Drug Registry.

### Other variables

Leisure time physical activity was based on reported minutes per week spent on 17 activities and activity-specific intensity factors. Smoking was defined as current, former and never. Alcohol consumption was defined as zero consumption (based on 7-day record and lifestyle questionnaire) and low (< 15 g/day, < 20 g/day), medium (15–30 g/day, 20–40 g/day) or high (> 30 g/day, > 40 g/day) in men and women respectively during the 7-day record. Highest level of education was defined as ≤ 8 years, 9–10 years, 11–13 years or university degree. Dietary change in the past (yes/no) was based on the question “Have you substantially changed your eating habits because of illness or other reasons?”

### Statistical analysis

The SPSS statistical computer package (version 20.0; IBM Corporation, Armonk, NY, USA) was used for statistical analyses.

We examined baseline characteristics across categories of the GRS and DRS, and in cases and non-cases of T2D, with the general linear model for continuous variables (adjusted for age and sex) and with chi^2^ test for categorical variables. Pearson correlation coefficients between energy-adjusted intakes of the foods in the DRS were computed.

We used Cox proportional hazards regression model to estimate hazard ratios (HRs) of diabetes incidence associated with tertiles of the GRS and three groups of the DRS for T2D, as well as for each dietary intake included in the DRS (adjusted for energy intake using the residual method). Years of follow-up were used as underlying time variable. In order to assess the proportional hazards assumption, we used graphs and tested interactions between the underlying time variable and examined covariates. The assumption was considered to be satisfied for all covariates except age. The presented results are therefore from age-stratified cox models (per 1-year age interval).

The basic model included adjustments for sex (when applicable). Our presented full multivariate model additionally included adjustments for BMI and total energy intake as continuous variables and for the following categorical variables: diet assessment method version, season of diet data collection, leisure time physical activity, smoking, alcohol intake and education. Missing data were treated as separate categories. Additional models included adjustment for intakes of fruit and vegetables, fermented dairy and high-fat fish, when applicable, but gave similar findings. We also performed analyses excluding BMI from the full multivariate model, as BMI may mediate associations between diet and T2D, but the results were virtually unchanged. Finally, we included waist or body fat percent instead of BMI in the multivariate model. Tests for interaction between the GRS and diet were performed, both by introducing multiplicative factors of the tertiles [GRS tertile × diet tertile (treated as continuous variables)]. Tertiles were used in order to overcome problems with outliers and to minimize power issues due to small groups when ranking the individual according to both diet and genetics. However, to test a more sensitive model, we also examined interaction between continuous variables.

In sensitivity analysis, we excluded individuals with dietary change in the past (22% of the individuals). In a second sensitivity analysis, we excluded individuals with prevalent cardiovascular disease (coronary event or stroke) at baseline (3%). All statistical tests were two-sided, and statistical significance was assumed at *P* < 0.05.

## Results

### Baseline characteristics

The baseline levels of established risk factors for T2D, as well as potential confounders of dietary associations, differed between incident cases and non-cases of T2D (Additional file 1: Table S2). Individuals who developed diabetes during follow-up were older and had higher fasting levels of glucose and insulin, higher BMI, a more sedentary lifestyle, higher protein intake and lower fibre intake. In addition, among incident cases of T2D, there were more males, ever-smokers as well as individuals reporting dietary change in the past and fewer individuals with high level of education. Fasting blood glucose increased across the GRS tertiles (Table 1), and several risk factors for T2D and potential confounders differed across the DRS tertiles. The dietary factors included in the DRS did not differ across tertiles of the GRS. Moreover, correlations between the four factors included in the DRS were very weak (Additional file 1: Table S3).

**Table 1:** Means (and standard deviations) or percentage distribution for baseline characteristics across tertiles of a weighted genetic risk score for type 2 diabetes and across categories of a diet risk score in 15,380 women and 9689 men from the Malmö Diet and Cancer Study

		All Tertile of genetic risk score				Women Tertile of genetic risk score				Men Tertile of genetic risk score			
	*n*	1	2	3	*P* trend*	1	2	3	*P* trend*	1	2	3	*P* trend*
Age (years)	25,069	58.3 (7.8)	58.2 (7.6)	58.2 (7.7)	0.32	57.3 (8.0)	57.3 (7.9)	57.3 (8.0)	0.86	59.5 (7.2)	59.0 (7.1)	59.1 (7.1)	0.04
BMI	25,035	25.7 (4.0)	25.7 (3.9)	25.6 (3.8)	0.12	25.3 (4.2)	25.4 (4.2)	25.3 4.0)	0.48	26.2 (3.5)	26.2 (3.4)	26.0 (3.4)	0.046
Fasting blood glucose (mmol/L)	4860	4.90 (0.6)	5.00 (0.7)	5.05 (0.8)	< 0.001	4.78 (0.5)	4.88 (0.6)	4.94 (0.7)	< 0.001	5.06 (0.6)	5.17 (0.8)	5.21 (1.0)	0.001
Fasting plasma insulin (mIU/L)	4819	7.8 (8.9)	7.7 (8.1)	7.7 (6.1)	0.89	7.3 (8.3)	7.0 (5.0)	7.2 (4.8)	0.75	8.5 (9.5)	8.7 (11.3)	8.6 (7.4)	0.93
HOMA-IR^†^	4571	1.60 (1.43)	1.59 (1.12)	1.61 (1.13)	0.76^†^	1.49 (1.54)	1.47 (1.04)	1.49 (0.96)	0.66^†^	1.76 (1.23)	1.77 (1.21)	1.80 (1.32)	0.29^†^
Alcohol intake (g/day) ^‡^	23,551	11.6 (12.8)	11.5 (12.9)	11.3 (12.3)	0.11	8.5 (8.9)	8.3 (8.6)	8.3 (8.7)	0.21	16.4 (16.0)	16.4 (16.4)	15.9 (15.3)	0.26
Processed meat (g/day)	25,069	38 (30)	38 (30)	38 (30)	0.61	31 (23)	31 (23)	31 (23)	0.57	51 (35)	49 (36)	50 (36)	0.68
Sugar-sweetened beverages (g/day)	25,069	76 (145)	78 (150)	77 (145)	0.64	67 (125)	65 (121)	66 (127)	0.83	91 (171)	99 (187)	95 (170)	0.42
Whole grain (portions/day)	25,069	1.0 (1.0)	1.0 (1.0)	1.0 (1.0)	0.93	0.9 (0.9)	0.9 (0.9)	0.9 (0.9)	0.65	1.0 (1.2)	1.1 (1.2)	1.1 (1.2)	0.58
Coffee (g/day)	25,069	520 (390)	530 (400)	510 (400)	0.77	510 (380)	520 (390)	510 (390)	0.87	530 (400)	540 (420)	530 (410)	0.77
					*P* value				*P* value				*P* value
Smoking (ever) (%)	25,059	62.4	61.7	61.1	0.22	56.7	55.6	54.9	0.17	71.3	71.3	71.4	0.99
Leisure time physical activity, high (%)^§^	24,913	18.9	20.6	20.2	0.02	19.4	20.1	20.2	0.58	18.2	21.4	20.3	0.01
Education (> 10 years) (%)	25,008	32.0	32.7	31.5	0.25	31.1	30.5	30.3	0.68	33.3	36.3	33.3	0.02
		Diet risk score				Diet risk score				Diet risk score			
	*n*	Low	Mid	High	*P* trend*	Low	Mid	High	*P* trend*	Low	Mid	High	*P* trend*
*n*		5699	15,188	4182		3767	9205	2408		1932	5983	1774	
Age (years)	25,069	57.6 (7.4)	58.4 (7.7)	58.7 (8.0)	< 0.001	56.6 (7.5)	57.4 (8.0)	57.8 (8.4)	< 0.001	58.6 (6.8)	59.3 (7.1)	59.6 (7.2)	< 0.001
BMI	25,035	25.4 (3.8)	25.6 (3.9)	26.0 (4.0)	< 0.001	25.0 (3.9)	25.3 (4.2)	25.7 (4.2)	< 0.001	26.1 (3.3)	26.1 (3.4))	26.6 (3.5)	< 0.001
Fasting blood glucose (mmol/L)	4860	4.94 (0.7)	4.98 (0.7)	5.07 (0.9)	< 0.001	4.82 (0.5)	4.89 (0.6)	4.92 (0.7)	0.005	5.13 (0.9)	5.12 (0.7)	5.28 (1.0)	0.03
Fasting plasma insulin (mIU/L)	4819	7.4 (7.3)	7.7 (8.0)	8.7 (7.6)	0.001	7.0 (8.1)	7.1 (5.4)	7.7 (4.8)	0.12	7.8 (5.4)	8.6 (10.6)	10.0 (9.8)	0.002
HOMA-IR^†^	4571	1.52 (1.49)	1.58 (1.07)	1.81 (1.35)	< 0.001	1.46 (1.68)	1.46 (0.91)	1.66 (1.14)	< 0.001	1.61 (1.04)	1.77 (1.23)	2.03 (1.54)	< 0.001
Alcohol intake (g/day)^‡^	23,551	11.5 (12.0)	11.4 (12.6)	11.7 (13.8)	0.53	8.6 (8.5)	8.4 (8.7)	7.8 (8.9)	0.007	15.9 (15.7)	16.0 (15.7)	17.4 (16.8)	0.009
Processed meat (g/day)	25,069	22 (21)	40 (30)	55 (31)	< 0.001	18 (16)	32 (23)	46 (24)	< 0.001	28 (26)	51 (36)	69 (36)	< 0.001
Sugar-sweetened beverages (g/day)	25,069	18 (70)	70 (140)	170 (200)	< 0.001	12 (52)	66 (124)	150 (156)	< 0.001	25 (91)	84 (161)	209 (235)	< 0.001
Whole grain (portions/day)	25,069	1.6 (1.2)	0.9 (0.9)	0.4 (0.5)	< 0.001	1.5 (0.9)	0.8 (0.8)	0.4 (0.4)	< 0.001	1.9 (1.4)	1.0 (1.1)	0.4 (0.5)	< 0.001
Coffee (g/day)	25,069	700 (410)	510 (390)	310 (260)	< 0.001	680 (400)	490 (370)	310 (270)	< 0.001	735 (430)	530 (410)	320 (260)	< 0.001
					*P* value				*P* value				*P* value
Smoking (ever) (%)	25,059	65.2	61.3	58.5	< 0.001	60.7	55.0	50.6	< 0.001	74.1	71.1	69.3	< 0.004
Leisure time Physical Activity, high (%)^§^	24,913	22.3	19.3	18.9	< 0.001	22.9	19.1	18.1	< 0.001	21.2	19.5	19.8	0.30
Education (> 10 years) (%)	25,008	37.7	31.6	26.3	< 0.001	35.7	30.4	23.6	< 0.001	41.8	33.3	29.9	< 0.001

*Adjusted for age and sex when applicable

^†^*P* value for ln transformed variables

^‡^Among those reporting that they consumed alcohol during the year before baseline examinations

^§^Highest quintile of leisure time physical activity

### The GRS and T2D

The GRS, designed based on previous GWAS findings, associated as expected with increased incidence of T2D (*P* for trend = 7 × 10^−35^), with a HR for individuals in the highest tertile of the GRS of 1.67 (95% CI 1.54, 1.81), compared with individuals in the lowest tertile. Similar observations were made in both genders (Table 2).

**Table 2:** Hazard ratios of incident type 2 diabetes according to a genetic risk score, and according to a diet risk score and its components, in 15,380 women and 9689 men from the Malmö Diet and Cancer Study

	All				Women				Men			
		Tertile*				Tertile*				Tertile*		
	1	2	3	*P* value for trend^†^	1	2	3	*P* value for trend^†^	1	2	3	*P* value for trend^†^
Cases/person-years	929/147393	1188/144429	1471/142031		478/92494	602/91511	764/91595		451/54897	586/52917	707/50436	
Genetic risk score	1.00	1.31 1.21, 1.43	1.67 1.54, 1.81	7 × 10^−35^	1.00	1.28 1.13, 1.44	1.62 1.44, 1.81	1 × 10^−16^	1.00	1.36 1.20, 1.53	1.72 1.53, 1.94	9 × 10^−20^
Cases/person-years	673/102570	2194/262381	721/68902		350/69708	1154/164327	340/41567		323/32862	1040/98054	381/27336	
Diet risk score	1.00	1.19 1.09, 1.30	1.40 1.26, 1.56	6 × 10^−10^	1.00	1.33 1.18, 1.50	1.45 1.26, 1.71	3 × 10^−7^	1.00	1.07 0.95, 1.22	1.33 1.14, 1.55	0.0002
Processed meat	1.00	1.01 0.93, 1.10	1.11 1.03, 1.21	0.009	1.00	1.06 0.95, 1.19	1.12 1.01, 1.27	0.03	1.00	0.98 0.87, 1.11	1.11 0.98, 1.25	0.08
SSB	1.00	1.01 0.93, 1.10	1.13 1.05, 1.22	0.003	1.00	1.06 0.94, 1.20	1.12 1.01, 1.25	0.03	1.00	0.95 0.84, 1.07	1.14 1.01, 1.28	0.06
Whole grain	1.00	0.94 0.86, 1.01	0.89 0.82, 0.96	0.004	1.00	0.95 0.85, 1.05	0.90 0.80, 1.02	0.09	1.00	0.92 0.82, 1.03	0.87 0.77, 0.98	0.02
Coffee	1.00	0.87 0.81, 0.95	0.75 0.69, 0.81	1 × 10^−11^	1.00	0.78 0.70, 0.87	0.63 0.56, 0.71	2 × 10^−14^	1.00	0.97 0.87, 1.10	0.88 0.78, 0.99	0.04

*For the DRS the levels refer to low risk score (0–2 points), medium risk score (3–5 points) and high risk score (6–8 points) instead of tertiles

^†^Age-stratified model, adjusted for sex when applicable. Examination of dietary variables was also adjusted for diet method version, season, total energy intake, BMI, leisure time physical activity, alcohol intake, smoking and education

### The DRS and T2D

The DRS based on high intakes of processed meat and SSB, and on low intakes of whole grain and coffee, associated with increased incidence of T2D (*P* for trend = 6 × 10^−10^), with 40% risk increase in the highest tertile (95% CI 26–56%). No major gender differences were seen (Table 2).

### Components of the DRS and T2D

All components of the DRS showed significant associations with incident T2D; we observed increased incidences at high intakes of processed meat (HR in the highest tertile 1.11; 95% CI 1.03, 1.21; *P* for trend = 0.009) and SSB (HR in the highest tertile 1.13; 95% CI 1.05, 1.22; *P* for trend = 0.003) and decreased incidences at high intakes of whole grain (HR in the highest tertile 0.89; 95% CI 0.82, 0.96; *P* for trend = 0.004) and coffee (HR in the highest tertile 0.75; 95% CI 0.69, 0.81; *P* for trend = 1 × 10^−11^) (Table 2). Similar tendencies were seen independently of gender, although the inverse association with coffee intake was significantly stronger in women (*P* for interaction with sex = 6 × 10^−5^).

### Interaction between the GRS and the DRS

**Figure 1:**
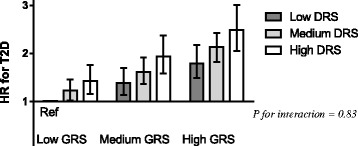
Hazard ratios of incident type 2 diabetes according to combinations of a genetic risk score and a dietary risk score for type 2 diabetes among individuals in the Malmö Diet and Cancer Study (*n* = 25,069). No statistical interaction was observed between the genetic and dietary risk scores (*P* = 0.83). Individuals with both high genetic susceptibility and unfavourable dietary habits had more than twice as high risk (HR 2.49; 95% CI 2.06, 3.01) of developing T2D compared to those with low genetic susceptibility and favourable dietary habits (reference HR = 1.00).

We did not observe any interaction between the GRS and the DRS on incidence of T2D (*P* = 0.83). The magnitude of the association between the GRS and T2D was similar at low, medium and high diet risk (Table 3), and likewise associations with the DRS did not differ depending on the genetic risk for T2D (Table 4). In joint effect model with low GRS and low DRS as reference, the highest risk estimate was seen for individuals with both high (3rd tertile) GRS and high DRS (HR 2.49; 95% CI 2.06, 3.01) (Fig. 1, Additional file 1: Table S4).

**Table 3:** Hazard ratios of incident type 2 diabetes according to a weighted genetic risk score in strata of a diet risk score based on intakes of processed meat, sugar-sweetened beverages (SSB), whole grain and coffee in 15,380 women and 9689 men from the Malmö Diet and Cancer Study

	All				Women				Men			
Diet risk score level/diet tertile	Tertile of genetic risk score			*P* value for trend*	Tertile of genetic risk score			*P* value for trend*	Tertile of genetic risk score			*P* value for trend*
	1	2	3		1	2	3		1	2	3	
Diet risk score												
Low	1.00	1.38 1.13, 1.68	1.71 1.41, 2.08	4 × 10^−8^	1.00	1.50 1.13, 1.99	1.75 1.34, 2.30	6 × 10^−5^	1.00	1.25 0.94, 1.68	1.69 1.28, 2.22	2 × 10^−4^
Medium	1.00	1.30 1.16, 1.45	1.66 1.49, 1.84	2 × 10^−21^	1.00	1.22 1.05, 1.43	1.62 1.40, 1.87	4 × 10^−11^	1.00	1.40 1.19, 1.64	1.71 1.47, 2.00	8 × 10^−12^
High	1.00	1.32 1.09, 1.60	1.69 1.41, 2.03	1 × 10^−8^	1.00	1.26 0.96, 1.67	1.50 1.16, 1.96	0.002	1.00	1.39 1.07, 1.83	1.85 1.44, 2.38	2 × 10^−6^
*P*_interaction_^†,‡^				0.83 (0.94)				0.40 (0.67)				0.60 (0.73)

*Age-stratified model, adjusted for sex when applicable

^†^Age-stratified model, adjusted for sex, diet method version, season, total energy intake, BMI, leisure time physical activity, alcohol intake, smoking and education

^‡^*P* for interaction treating tertiles as continuous variables and, in brackets, *P* for interaction between continuous variables of the GRS and the DRS

**Table 4:** Hazard ratios of incident type 2 diabetes according to a diet risk score based on intakes of processed meat, sugar-sweetened beverages (SSB), whole grain and coffee in strata of a weighted genetic risk score among 15,380 women and 9689 men from the Malmö Diet and Cancer Study

	All				Women				Men			
Diet risk score level/diet tertile	Tertile of genetic risk score				Tertile of genetic risk score				Tertile of genetic risk score			
	1	2	3		1	2	3		1	2	3	
Diet risk score												
Low	1.00	1.00	1.00		1.00	1.00	1.00		1.00	1.00	1.00	
Medium	1.23 1.03, 1.46	1.17 1.01, 1.36	1.20 1.04, 1.37		1.48 1.16, 1.90	1.23 1.00, 1.51	1.32 1.09, 1.59		1.02 0.80, 1.31	1.12 0.89, 1.39	1.11 0.91, 1.36	
High	1.42 1.15, 1.76	1.42 1.18, 1.71	1.40 1.18, 1.65		1.68 1.24, 2.27	1.45 1.11, 1.89	1.38 1.09, 1.76		1.21 0.90, 1.63	1.40 1.07, 1.83	1.40 1.10, 1.78	
*P*_trend_*	0.001	3 × 10^−4^	8 × 10^−5^		0.001	0.006	0.005		0.20	0.01	0.005	
*P*_interaction_*^,†^				0.83 (0.94)				0.40 (0.67)				0.60 (0.73)

*Age-stratified model, adjusted for sex, diet method version, season, total energy intake, BMI, leisure time physical activity, alcohol intake, smoking and education

^†^*P* for interaction treating tertiles as continuous variables and, in brackets, *P* for interaction between continuous variables of the GRS and the DRS

### Interaction between the GRS and components in the DRS

We did not observe any significant interactions between the components in the DRS and the GRS on incident T2D (Additional file 1: Table S5). Regarding whole grain intake and the GRS in gender-specific analyses, we observed some non-significant tendencies of interactions, although in opposite directions; in women, the magnitude of the inverse association between whole grain intake and T2D tended to decrease with higher genetic risk (*P* for interaction = 0.07), whereas it tended to increase with higher genetic risk in men (*P* for interaction = 0.07).

### Secondary analyses with extended DRS for T2D

Extended DRSs, that additionally included intakes (in tertiles) of fruit and vegetables, fermented dairy or high-fat fish showed similar associations with T2D as the original DRS (Additional file 1: Table S6), and intakes of the additional food components in the extended scores did not show significant associations with T2D in gender-combined analyses, favouring our approach of not including these foods in the initial diet score. However, in women, fermented dairy intake was inversely associated with T2D (*P* for trend = 0.002) (*P* for interaction with sex = 0.02).

Furthermore, we did not observe any interactions between the GRS and the extended DRSs (Additional file 1: Table S7) (all *P* values for interactions ≥ 0.16) or their added food components (Additional file 1: Table S8), in gender-combined analyses. In women, our results suggested some possible, although unclear, modification by the GRS for the role of fruit and vegetable intake in T2D incidence (*P* for interaction = 0.04), and we found a non-significant tendency of stronger inverse association between fermented dairy and T2D among women with lower GRS (*P* for interaction = 0.07).

### Secondary analyses in a subsample with extended GRS for T2D

In the subsample of the MDC study (*n* = 4193), with genetic data on 68 T2D associated SNPs, no statistical interaction was observed between the extended GRS and the DRS (*P* = 0.34) (Additional file 2: Figure S1). Higher DRS associated with an increased risk of T2D in the low (*P* for trend across DRS tertiles = 0.001), medium (*P* for trend = 0.003) and high tertile (*P* for trend = 0.04) of the extended GRS. Individuals with both high genetic susceptibility and unfavourable dietary habits had more than twice as high risk (HR 3.82; 95% CI 2.18, 6.71) of developing T2D compared to those with low genetic susceptibility and favourable dietary habits (reference HR = 1.00).

### Statistical models with waist circumference and body fat percent

Replacing BMI with waist or body fat percent in our multivariate models did not substantially change our observations. However, no tendencies of interactions between whole-grain intake and the GRS remained in the gender-specific analyses, when waist or body fat percent replaced BMI.

### Sensitivity analysis

After excluding individuals reporting dietary change in the past, our results remained virtually unchanged, indicating that unfavourable diet and genetic predisposition independently contribute to an increased risk of T2D. Excluding individuals with prevalent cardiovascular disease did not either change our findings (data not shown).

## Discussion

**Figure 2:**
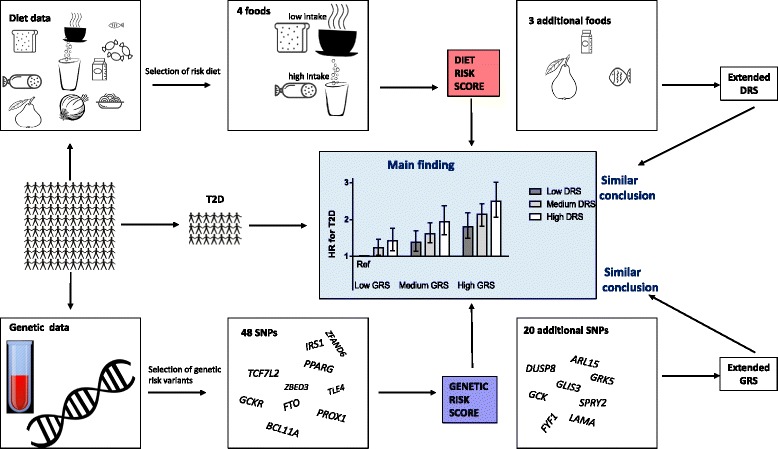
The positive association of similar magnitude independent of genetic predisposition to T2D, assessed using a GRS composed of 48 SNPs identified in GWAS for T2D

A risk score, of food intakes consistently associated with risk of T2D (processed meat, SSB, whole grain and coffee), was in our population-based prospective study associated with increased incidence of T2D. The positive association was of similar magnitude independent of genetic predisposition to T2D, assessed using a GRS composed of 48 SNPs identified in GWAS for T2D (Fig. 2). Likewise, each food component of the DRS associated with incidence of T2D, independently of the GRS. The highest risk of T2D was seen in individuals with both high genetic and dietary risk scores. Adding foods less consistently associated with T2D, in scientific literature, to create an extended DRS did not importantly alter the findings regarding the original DRS, and lack of overall association between these foods and T2D in our study supports the initial approach of not including them in the DRS.

To the best of our knowledge, no study has previously examined if a GRS for T2D modifies associations between a T2D-specific dietary risk pattern and incidence of T2D. Similar to our findings, no interaction was observed between a GRS for T2D and dietary habits assessed by a Mediterranean diet score [31]. In line with this, an overall healthy diet score associated with lower fasting glucose and fasting insulin independently of genotypes previously associated with glucose homeostasis [45]. However, in US men, a higher score of a GRS, based on 10 T2D-associated SNPs, was found to accentuate the increased risk of T2D associated with a Western dietary pattern, characterized by meat, refined cereals, sweets and desserts [46]. Moreover, genetic variations in the *FTO* and *MC4R* genes have been reported to interact with a Mediterranean diet score on T2D [47], and a genetic variation in *ADRA2B* has been reported to interact with diet quality, based on fat and fibre intakes, on T2D [48]. Few results regarding interactions between GRSs and specific dietary components on T2D or related traits have been reported; no interaction was detected between a GRS of 15 SNPs and carbohydrates or fibre in the National Health and Nutrition Examination Survey [49], and meat intake was found to associate with fasting concentrations of glucose and insulin independently of a GRS in a meta-analysis of 14 cohorts [50]. Most indications of interactions between dietary factors and genetic variants have come off from investigations of single T2D SNPs and specific dietary factors [2, 3]. Regarding the components included in the DRS of our study and earlier reported interactions with T2D associated SNPs, fibre or whole-grain intakes have been found to interact with genetic variation in *TCF7L2*, by us and others, as well as with variation in *NOTCH2* and *ZBED3* [2, 51, 52]. In addition, interaction between coffee consumption and genetic variation in *CDCAL1* and *IGF2BP2* has been reported [53].

The 6% of the individuals in our study, categorized as having both high genetic susceptibility and unfavourable dietary habits, were found to have twice until up to three times as high risk compared to those with low genetic susceptibility and favourable dietary habits. Our findings also show that dietary habits previously associated with T2D are of importance in the prevention of the disease independently of an individual’s genetic susceptibility, as the magnitude of our observed relative risk decrease by favourable diet was similar in individuals with high and low GRS. Still, it is important to consider that a 30% risk decrease, as observed at a low compared with a high DRS in our study, would be more important for individuals with higher genetic susceptibility to T2D, as the absolute risk decrease would be greater (i.e. a decrease from about 10 to 7 incident cases per 1000 person-years in the highest GRS tertile, compared with a decrease from 6 to 4 cases in the lowest GRS tertile), meaning that healthy dietary habits should be especially crucial for individuals genetically predisposed to the disease.

Regarding the observed tendencies of interactions between the GRS and whole grain in the gender-specific analyses, we are prone to believe that the inconsistent associations between whole grain and T2D in strata of the GRS may have occurred due to chance, or loss of power when stratifying on both gender and GRS, especially since we observed tendencies of interactions in opposite directions in women and men and since those tendencies disappeared when waist or body fat percent replaced BMI in the statistical models.

We examined interaction between a DRS of known dietary T2D risk factors and a GRS of GWAS-identified T2D SNPs. However, it is possible that the foods showing consistent overall associations with T2D are repeatedly identified as risk factors due to low degree of interaction with other factors, such as genetics. Indeed, our results regarding foods previously showing less consistent associations with T2D, such as intakes of fruit and vegetables, indicated some putative interactions, although restricted to women. It remains to be examined if other food intakes not previously associated with T2D may be identified in subgroups depending on genetic risk. Besides, genome-wide interaction studies could be designed to identify new genetic loci interacting with dietary factors [54, 55], because the genetic variants that are most likely to interact with lifestyle factors may not be identified in conventional GWAS, as such variants may only associate with T2D in subgroups of individuals that are similar with regard to certain lifestyle factors [56, 57]. Finally, as we eat a mix of foods or nutrients and as dietary factors may interact with each other, it is from a public health point of view crucial to examine if overall genetic predisposition to disease modifies the importance of overall dietary patterns. Nevertheless, as different food components and variations in different genetic loci are critical in disease development via various mechanisms, genetic variants and dietary factors involved in the same biological pathways are more likely to interact. Consequently, the fact that most T2D SNPs associate with beta cell dysfunction and thus insulin secretion, while dietary factors may more likely associate with insulin resistance, could partly explain the lack of interaction between the GRS and the DRS. Whether our DRS for T2D and its included components interact with specific T2D loci was out of the scope of the present study. Despite lack of interaction between diet and accumulated genetic risk for T2D, dietary factors may still be more or less important depending on whether we carry single genetic risk variants that interact with those dietary factors, and therefore, our results do not contradict previous studies indicating that interactions between specific loci and individual dietary components exist. However, well-powered studies with good-quality dietary data are needed to replicate findings of both kinds [58].

Our study has several strengths that can be emphasized. Firstly, it is a large study with long follow-up time. Second, due to the population-based prospective design, selection bias and reverse causation should be minor issues. Third, we have extensive information on potential confounders. Fourth, diet data were of high quality [37, 38] and the foods included in the DRS clearly associated with T2D in expected directions. Moreover, we had the possibility to exclude individuals with reported dietary changes in the past. Still, it is a limitation that diet was only measured at baseline. Moreover, we did not have genotype information on T2D SNPs identified after the publication by Morris et al. in the whole study sample. However, our main finding persisted similar in the analysis of a subsample including 17% of the individuals with genotype data on 20 additional T2D SNPs. Our focus on overall genetic and diet risks may also be a limitation, and future studies could aim at constructing scores based on functional annotations. Further, due to lack of sufficient scientific evidence, we were not able to construct a weighted diet risk score, although some of the dietary factors may be especially crucial with regard to T2D development. Finally, we cannot exclude occurrence of residual confounding.

## Conclusions

Our findings show that both the dietary and genetic risk factors examined in this study add to the risk of T2D; the highest risk was seen in individuals with high scores for both factors. The study supports the view that overall dietary and genetic risk contribute to the disease in an independent fashion and that all individuals, whether at high or low genetic risk, may benefit from favourable dietary habits. However, it may be essential to consider that a similar relative decrease in risk is of greater value to individuals already at high genetic risk.

## Additional files

Additional file 1: **Table S1.** Single nucleotide polymorphisms included in the GRS as they were reported to associate with type 2 diabetes by Morris et al. **Table S2.** Baseline characteristics in 25,069 cases and non-cases of incident type 2 diabetes from Malmö diet and cancer. **Table S3.** Correlation coefficients^a^ between energy-adjusted intakes of components in a diet risk score for type 2 diabetes in 25,069 individuals from Malmö diet and cancer. **Table S4.** Hazard ratios of incident type 2 diabetes according to combinations of a weighted genetic risk score and a diet risk score based on intakes of processed meat, sugar-sweetened beverages (SSB), whole grain and coffee in 15,380 women and 9689 men from Malmö diet and cancer. **Table S5.** HR of incident type 2 diabetes according to combinations of a weighted genetic risk score and components of a diet risk score in 15,380 women and 9689 men from Malmö diet and cancer. **Table S6.** HR^a^ of incident type 2 diabetes according to extended dietary risk scores (DRS) for type 2 diabetes and the added dietary components in 15,380 women and 9689 men from Malmö diet and cancer. **Table S7.** HR of incident type 2 diabetes according to tertiles of a genetic risk score and alternative dietary risk scores (DRS) including additional diet components in 15,380 women and 9689 men from Malmö diet and cancer. **Table S8.** HR of incident type 2 diabetes (T2D) according to tertiles of a genetic risk score and intakes of the additional components in the alternative dietary risk scores in 15,380 women and 9689 men from Malmö diet and cancer. **Table S9.** Additional single nucleotide polymorphisms included in the extended GRS and reported to associate with type 2 diabetes by Fuchsberger et al. 2016. (DOCX 116 kb)

Additional file 2: **Figure S1.** In a subsample (*n* = 4193), with genetic data on 68 T2D SNPs (20 additional SNPs), the findings were similar to those in the whole study sample. No statistical interaction was observed between the extended genetic risk score and the dietary risk scores (*P* = 0.34). Individuals with both high genetic susceptibility and unfavourable dietary habits had more than twice as high risk (HR: 3.82; 95% CI: 2.18, 6.71) of developing T2D compared to those with low genetic susceptibility and favourable dietary habits (reference HR = 1.00). (DOCX 37 kb)

## Abbreviations

DRS: Diet risk score
GRS: Genetic risk score
MDC: Malmö Diet and Cancer
T2D: Type 2 diabetes
