# Supplementary material for: Dietary and genetic risk scores and incidence of type 2 diabetes
Source: Genes Nutr. 2018 May 16;13:13. doi: 10.1186/s12263-018-0599-1 (PMC5956794; doi:10.1186/s12263-018-0599-1)
Supplement: Supplementary file 1 — Table S1. Single nucleotide polymorphisms included in the GRS as they were reported to associate with type 2 diabetes by Morris et al. Table S2. Baseline characteristics in 25,069 cases and non-cases of incident type 2 diabetes from Malmö diet and cancer. Table S3. Correlation coefficientsa between energy-adjusted intakes of components in a diet risk score for type 2 diabetes in 25,069 individuals from Malmö diet and cancer. Table S4. Hazard ratios of incident type 2 diabetes according to combinations of a weighted genetic risk score and a diet risk score based on intakes of processed meat, sugar-sweetened beverages (SSB), whole grain and coffee in 15,380 women and 9689 men from Malmö diet and cancer. Table S5. HR of incident type 2 diabetes according to combinations of a weighted genetic risk score and components of a diet risk score in 15,380 women and 9689 men from Malmö diet and cancer. Table S6. HRa of incident type 2 diabetes according to extended dietary risk scores (DRS) for type 2 diabetes and the added dietary components in 15,380 women and 9689 men from Malmö diet and cancer. Table S7. HR of incident type 2 diabetes according to tertiles of a genetic risk score and alternative dietary risk scores (DRS) including additional diet components in 15,380 women and 9689 men from Malmö diet and cancer. Table S8. HR of incident type 2 diabetes (T2D) according to tertiles of a genetic risk score and intakes of the additional components in the alternative dietary risk scores in 15,380 women and 9689 men from Malmö diet and cancer. Table S9. Additional single nucleotide polymorphisms included in the extended GRS and reported to associate with type 2 diabetes by Fuchsberger et al. 2016. (DOCX 116 kb) [file 12263_2018_599_MOESM1_ESM.docx]

**Table S1 - Single nucleotide polymorphisms included in the GRS as they were reported to associate with type 2 diabetes by Morris et al.**

| SNP | Chromosome: position* | Location | Minor  Allele Frequency | Minor allele | Risk allele | Ref.  Including  original ref. | References |
| --- | --- | --- | --- | --- | --- | --- | --- |
| 1. IRS1 rs2943641 | 2:226229029 | intergenic | 0.36 | T | C | [1]  [2] | Morris AP, Voight BF, Teslovich TM, Ferreira T, Segre AV, Steinthorsdottir V, Strawbridge RJ, Khan H, Grallert H, Mahajan A, et al: **Large-scale association analysis provides insights into the genetic architecture and pathophysiology of type 2 diabetes.** *Nat Genet* 2012, **44:**981-990.  Rung J, Cauchi S, Albrechtsen A, Shen L, Rocheleau G, Cavalcanti-Proenca C, Bacot F, Balkau B, Belisle A, Borch-Johnsen K, et al: **Genetic variant near IRS1 is associated with type 2 diabetes, insulin resistance and hyperinsulinemia.** *Nat Genet* 2009, **41:**1110-1115 |
| 1. PPARG rs1801282 | 3:12351626 | exon, missense | 0.15 | G | C | [1]  [3] | Diabetes Genetics Initiative of Broad Institute of H, Mit LU, Novartis Institutes of BioMedical R, Saxena R, Voight BF, Lyssenko V, Burtt NP, de Bakker PI, Chen H, Roix JJ, et al: **Genome-wide association analysis identifies loci for type 2 diabetes and triglyceride levels.** *Science* 2007, **316:**1331-1336. |
| 1. TCF7L2 rs7903146 | 10:112998590 | intron |  |  |  | [1,3] |  |
| 1. FTO rs9939609 | 16:53786615 | intron | 0.26 | T | T | [1]  [4] | Frayling TM, Timpson NJ, Weedon MN, Zeggini E, Freathy RM, Lindgren CM, Perry JR, Elliott KS, Lango H, Rayner NW, et al: **A common variant in the FTO gene is associated with body mass index and predisposes to childhood and adult obesity.** *Science* 2007, **316:**889-894. |
| 1. BCL11A rs243021 | 2:60357684 | intergenic | 0.46 | T | T | [1]  [5] | Voight BF, Scott LJ, Steinthorsdottir V, Morris AP, Dina C, Welch RP, Zeggini E, Huth C, Aulchenko YS, Thorleifsson G, et al: **Twelve type 2 diabetes susceptibility loci identified through large-scale association analysis.** *Nature genetics* 2010, **42:**579-589. |
| 1. ZBED3 rs4457053 | 5:77129124 | intron | 0.26 | G | G | [1,5] |  |
| 1. ZFAND6 rs11634397 | 15:80139880 | intergenic | 0.40 | A | G | [1,5] |  |
| 1. TLE4 rs13292136 | 9:79337213 | intergenic | 0.07 | T | C | [1,5] |  |
| 1. HMGA2 rs1531343 | 12:65781114 | intron | 0.90 | C | C | [1,5] |  |
| 1. ARAP1 rs1552224 | 11:72722053 | 5´UTR | 0.12 | G | T | [1,5] |  |
| 1. DGKB rs2191349 | 7:15024684 | intergenic | 0.48 | G | T | [1]  [6] | Dupuis J, Langenberg C, Prokopenko I, Saxena R, Soranzo N, Jackson AU, Wheeler E, Glazer NL, Bouatia-Naji N, Gloyn AL, et al: **New genetic loci implicated in fasting glucose homeostasis and their impact on type 2 diabetes risk.** *Nature genetics* 2010, **42:**105-116. |
| 1. PROX1 rs340874 | 1:213985913 | intron | 0.48 | A | G | [1,6] |  |
| 1. GCKR rs780094 | 2:27518370 | intron | 0.48 | A | G | [1,6] |  |
| 1. HNF1A rs7957197 | 12:121022883 | intron | 0.15 | A | T | [1,5] |  |
| 1. PRC1 rs8042680 | 15:90978107 | intron | 0.22 | A | A | [1,5] |  |
| 1. TP53INP1 rs896854 | 8:94948283 | intron | 0.48 | A | A | [1,5] |  |
| 1. KLF14 rs972283 | 7:130782095 | intron | 0.45 | A | G | [1,5] |  |
| 1. RBMS1 rs7593730 | 2:160314943 | intron | 0.33 | T | C | [1]  [7] | Qi L, Cornelis MC, Kraft P, Stanya KJ, Linda Kao WH, Pankow JS, Dupuis J, Florez JC, Fox CS, Pare G, et al: **Genetic variants at 2q24 are associated with susceptibility to type 2 diabetes.** *Hum Mol Genet* 2010, **19:**2706-2715. |
| 1. KIAA1486/ IRS1 rs7578326 | 2:226155937 | intron | 0.36 | G | A | [1,5] |  |
| 1. WFS1 rs10010131 | 4:6293966 | intron | 0.40 | A | G | [1]  [8] | Sandhu MS, Weedon MN, Fawcett KA, Wasson J, Debenham SL, Daly A, Lango H, Frayling TM, Neumann RJ, Sherva R, et al: **Common variants in WFS1 confer risk of type 2 diabetes.** *Nat Genet* 2007, **39:**951-953. |
| 1. JAZF1 rs864745 | 7:28140937 | intron | 0.499 | G | A | [1]  [9] | Zeggini E, Scott LJ, Saxena R, Voight BF, Marchini JL, Hu T, de Bakker PI, Abecasis GR, Almgren P, Andersen G, et al: **Meta-analysis of genome-wide association data and large-scale replication identifies additional susceptibility loci for type 2 diabetes.** *Nat Genet* 2008, **40:**638-645. |
| 1. SLC30A8 rs13266634 | 8:117172544 | intron | 0.35 | T | C | [1,3] |  |
| 1. CDKAL1 rs7754840 | 6:20660803 | intron | 0.31 | C | C | [1,3] |  |
| 1. ADAMTS9 rs4607103 | 3:64726228 | intron | 0.24 | T | C | [1,9] |  |
| 1. CAMK1D rs12779790 | 10:12286011 | intergenic | 0.18 | G | G | [1,9] |  |
| 1. CDKNA2B rs10811661 | 9:22134095 | intergenic | 0.17 | C | T | [1,3] |  |
| 1. HHEX rs1111875 | 10:92703125 | intergenic | 0.47 | A | G | [1,3] |  |
| 1. KCNJ11 rs5219 | 11:17388025 | exon, missense | 0.47 | A | A | [1]  [10] | Gloyn AL, Weedon MN, Owen KR, Turner MJ, Knight BA, Hitman G, Walker M, Levy JC, Sampson M, Halford S, et al: **Large-scale association studies of variants in genes encoding the pancreatic beta-cell KATP channel subunits Kir6.2 (KCNJ11) and SUR1 (ABCC8) confirm that the KCNJ11 E23K variant is associated with type 2 diabetes.** *Diabetes* 2003, **52:**568-572. |
| 1. DCD rs1153188 | 12:54705212 | intergenic | 0.27 | A | A | [1,9] |  |
| 1. MTNR1B rs10830963 | 11:92975544 | intron | 0.30 | G | G | [1,6] |  |
| 1. NOTCH2 rs10923931 | 1:119975336 | intron | 0.11 | T | T | [1,9] |  |
| 1. THADA rs7578597 | 2:43505684 | Exon,  missense | 0.10 | C | T | [1,9] |  |
| 1. TSPAN8 rs7961581 | 12:71269322 | intergenic | 0.27 | C | C | [1,9] |  |
| 1. KCNQ1 rs2237895 | 11:2,835,964 | intron | 0.40 | C | C | [1]  [11] | Unoki H, Takahashi A, Kawaguchi T, Hara K, Horikoshi M, Andersen G, Ng DP, Holmkvist J, Borch-Johnsen K, Jorgensen T, et al: **SNPs in KCNQ1 are associated with susceptibility to type 2 diabetes in East Asian and European populations.** *Nat Genet* 2008, **40:**1098-1102. |
| 1. ADCY5 rs11708067 | 3:123346931 | intron | 0.22 | G | A | [1,6] |  |
| 1. IGF2BP2 rs4402960 | 3:185,793,899 | intron | 0.30 | C | C | [1,3] |  |
| 1. VEGFA rs9472138 | 6:43844025 | intron | 0.28 | T | T | [1,9] |  |
| 1. CILP2 rs10401969 | 19:19296909 | intron | 0.08 | C | C | [1] |  |
| 1. KLHDC5 rs10842994 | 12:27812217 | intron | 0.20 | T | C | [1] |  |
| 1. ZMIZ1 rs12571751 | 10:79182874 | intron | 0.48 | G | A | [1] |  |
| 1. MC4R rs12970134 | 18:60217517 | intergenic | 0.27 | A | A | [1] |  |
| 1. GRB14 rs13389219 | 2:164672366 | intergenic | 0.40 | T | C | [1] |  |
| 1. ANKRD55 rs459193 | 5:56510924 | intron | 0.30 | A | G | [1] |  |
| 1. ANK1 rs516946 | 8:41661730 | intron | 0.24 | T | C | [1] |  |
| 1. HNF1B rs7501939 | 17:37741165 | intron | 0.39 | T | T | [1,5] |  |
| 1. HMG20A rs7177055 | 15:77540420 | intergenic | 0.32 | G | A | [1] |  |
| 1. BCAR1/ CTRB2 rs7202877 | 16:75213347 | intergenic | 0.11 | G | T | [1] |  |
| 1. KCNQ1 rs231362 | 11:2670241 | intron | 0.48 | A | A | [1,5] |  |

*According to the human reference genome *GRCh38*, released from the [Genome Reference Consortium](https://en.wikipedia.org/wiki/Genome_Reference_Consortium) 2013

**Table S2 - Baseline characteristics in 25,069 cases and non-cases of incident type 2 diabetes from the Malmö Diet and Cancer study**

|  |  | Means (95% CI) or percentage distribution | | | | | | | | |
| --- | --- | --- | --- | --- | --- | --- | --- | --- | --- | --- |
|  |  | All | | | Women | | | Men | | |
| Baseline variable | n | Cases  (n=3,588) | Non-cases  (n=21,481) | P value ^a^ | Cases  (n=1,844) | Non-cases  (n=13,536) | P value ^a^ | Cases  (n=1,744) | Non-cases  (n=7,945) | P value ^a^ |
| Sex (%women) | 25 069 | 51.4% | 63.0% | <0.001 |  |  |  |  |  |  |
| Age (y) | 25 069 | 58.2  58.0, 58.4 | 58.2  58.1, 58.3 | 0.77 | 57.8  57.4, 58.2 | 57.2  57.1, 57.4 | 0.003 | 58.6  58.3, 58.9 | 59.3  59.1, 59.5 | <0.001 |
| BMI (kg/m^2^) | 25 035 | 28.0  27.9, 28.1 | 25.3  25.3, 25.4 | <0.001 | 27.9  27.7, 28.1 | 25.0  24.9, 25.0 | <0.001 | 28.1  28.0, 28.3 | 25.7  25.7, 25.8 | <0.001 |
| Waist (cm) | 25 028 | 91.8  91.4, 92.2 | 84.4  84.2, 84.5 | <0.001 | 84.8  84.3, 85.4 | 76.6  76.4, 76.8 | <0.001 | 98.8  98.2, 99.4 | 92.3  92.1, 92.6 | <0.001 |
| Body fat (%) | 24 922 | 27.9  27.8, 28.1 | 25.2  25.2, 25.3 | <0.001 | 33.3  33.1, 33.5 | 30.3  30.2, 30.4 | <0.001 | 22.5  22.3, 22.7 | 20.2  20.1, 20.4 | <0.001 |
| Systolic blood pressure (mmHg) | 25 032 | 146  146, 147 | 140  140, 142 | <0.001 | 145  144, 145 | 138  138, 139 | <0.001 | 148  147, 149 | 143  142, 143 | <0.001 |
| Diastolic blood pressure (mmHg) | 25 030 | 88.7  88.4, 89.0 | 85.4  85.3, 85.5 | <0.001 | 86.9  86.4, 87.3 | 83.5  83.4, 83.7 | <0.001 | 90.4  90.0, 90.9 | 87.4  87.2, 87.6 | <0.001 |
| HbA1c (%) | 4 860 | 5.10  5.06, 5.13 | 4.75  4.73, 4.76 | <0.001 | 5.10  5.06, 5.14 | 4.77  4.75, 4.78 | <0.001 | 5.09  5.04, 5.14 | 4.72  4.70, 4.75 | <0.001 |
| Fasting blood glucose (mmol/L) | 4 860 | 5.68  5.64, 5.73 | 4.87  4.85, 4.90 | <0.001 | 5.51  5.46, 5.56 | 4.76  4.74, 4.78 | <0.001 | 5.86  5.79, 5.93 | 4.98  4.94, 5.01 | <0.001 |
| Triglycerides (mmol/L) | 4 867 | 1.68  1.63, 1.73 | 1.30  1.28, 1.32 | <0.001 | 1.57  1.52, 1.63 | 1.17  1.15, 1.19 | <0.001 | 1.79  1.70, 1.87 | 1.43  1.39, 1.48 | <0.001 |
| HDL-C (mmol/L) | 4 821 | 1.25  1.23, 1.87 | 1.38  1.37, 1.40 | <0.001 | 1.38  1.34, 1.41 | 1.54  1.52, 1.55 | <0.001 | 1.13  1.10, 1.16 | 1.23  1.22, 1.25 | <0.001 |
| LDL-C (mmol/L) | 4 764 | 4.29  4.22, 4.36 | 4.14  4.11, 4.17 | <0.001 | 4.39  4.29, 4.48 | 4.17  4.13, 4.21 | <0.001 | 4.18  4.09, 4.27 | 4.12  4.08, 4.17 | 0.26 |
| Fasting plasma insulin (mU/L) | 4 695 | 11.8  11.3, 12.4 | 7.1  6.8, 7.3 | <0.001 | 10.4  9.8, 10.9 | 6.6  6.3, 6.8 | <0.001 | 13.4  12.5, 14.4 | 7.5  7.0, 8.0 | <0.001 |
| HOMA-IR | 4 485 | 2.27  2.18, 2.37 | 1.53  1.49, 1.57 | <0.001^b^ | 2.05  1.92, 2.18 | 1.40  1.35, 1.45 | <0.001^b^ | 2.52  2.37, 2.67 | 1.65  1.59, 1.71 | <0.00^b^ |
| Leisure time Physical Activity Score^c^ | 24 913 | 7650  7430, 7870 | 8250  8160, 8350 | <0.001 | 7450  7160, 7750 | 8050  7940, 8160 | <0.001 | 7940  7600, 8270 | 8461  8310, 8620 | 0.005 |
| Alcohol intake (g/d)^d^ | 23 551 | 11.9  11.5, 12.3 | 12.4  12.3, 12.6 | 0.01 | 7.2  6.8, 7.6 | 8.5  8.4, 8.7 | <0.001 | 16.4  15.7, 17.1 | 16.2  15.9, 16.6 | 0.64 |
| Smoking (previous and current) (%) | 25 059 | 65.2% | 61.2% | <0.001 | 56.8% | 55.6% | 0.32 | 74.1% | 70.7% | 0.005 |
| Education (>10y) (%) | 25 008 | 26.0% | 33.1% | <0.001 | 23.0% | 31.7% | <0.001 | 29.1% | 35.5% | <0.001 |
| Dietary change in the past (%) | 25 035 | 26.6% | 21.5% | <0.001 | 29.0% | 22.7% | <0.001 | 24.1% | 19.3% | <0.001 |
| Energy (kcal/d) | 25 069 | 2330  2310, 2350 | 2350  2350, 2360 | 0.01 | 2010  1990, 2040 | 2040  2040, 2050 | 0.01 | 2640  2610, 2670 | 2660  2650, 2680 | 0.15 |
| Protein (E%) | 25 069 | 15.8  15.8, 15.9 | 15.4  15.4, 15.5 | <0.001 | 15.9  15.8, 16.0 | 15.6  15.5, 15.6 | <0.001 | 15.5  15.4, 15.7 | 15.1  15.0, 15.1 | <0.001 |
| Carbohydrates (E%) | 25 069 | 46.0  45.8, 46.2 | 46.3  46.2, 46.4 | 0.01 | 46.6  46.3, 46.9 | 46.7  46.6, 46.8 | 0.69 | 45.2  44.9, 45.5 | 45.7  45.6, 45.9 | 0.001 |
| Fat (E%) | 25 069 | 38.3  38.1, 38.5 | 38.5  38.4, 38.6 | 0.06 | 37.4  37.1, 37.6 | 37.8  37.6, 37.9 | 0.009 | 39.2  38.7, 39.5 | 39.2  39.0, 39.3 | 0.99 |
| Saturated fat (E%) | 25 069 | 16.2  16.1, 16.3 | 16.5  16.4, 16.5 | <0.001 | 16.0  15.9, 16.2 | 16.4  16.4, 16.5 | <0.001 | 16.6  16.4, 16.8 | 16.8  16.7, 16.8 | 0.19 |
| Fiber (g/1000kcal) | 25 069 | 9.1  9.0, 9.1 | 9.2  9.2, 9.3 | <0.001 | 9.6  9.5, 9.7 | 9.8  9.7, 9.8 | 0.006 | 8.4  8.3, 8.5 | 8.6  8.5, 8.6 | 0.02 |
| Processed meat (g/d) | 25 069 | 42  41, 43 | 39  38, 39 | <0.001 | 33  32, 34 | 30  30, 31 | <0.001 | 53  51, 54 | 50  49, 50 | <0.001 |
| Sugar-sweetened beverages (g/d) | 25 069 | 87  83, 92 | 78  76, 80 | <0.001 | 78  73, 84 | 66  64, 69 | <0.001 | 103  95,111 | 97  93, 101 | 0.18 |
| Whole grain products (portions/d) | 25 069 | 0.89  0.86, 0.93 | 0.96  0.95, 0.98 | <0.001 | 0.87  0.83, 0.91 | 0.92  0.90, 0.93 | 0.02 | 0.96  0.91, 1.01 | 1.05  1.02, 1.08 | 0.006 |
| Coffee (g/d) | 25 069 | 509  496, 522 | 521  515, 527 | 0.08 | 489  472, 507 | 512  505, 518 | 0.02 | 534  515, 553 | 536  526, 544 | 0.91 |
| Fruits (g/d) | 25 069 | 186  182, 190 | 187  185, 189 | 0.71 | 206  200, 211 | 209  207, 211 | 0.27 | 173  167, 179 | 171  167, 173 | 0.48 |
| Vegetables (g/d) | 25 069 | 176  173, 179 | 177  176, 179 | 0.39 | 187  183, 192 | 187  185-188 | 0.74 | 167  162, 172 | 171  169, 173 | 0.15 |
| Fermented dairy (portions/d) | 25 069 | 2.59  2.53, 2.64 | 2.65  2.62, 2.67 | 0.04 | 2.60  2.53, 2.68 | 2.76  2.73, 2.79 | <0.001 | 2.74  2.65, 2.82 | 2.68  2.64, 2.71 | 0.18 |
| High-fat fish (g/d) | 25 069 | 16.3  15.6, 16.9 | 15.8  15.5, 16.0 | 0.16 | 14.3  13.5, 15.1 | 13.8  13.5, 14.1 | 0.23 | 19.0  17.9, 20.2 | 18.5  17.9, 19.0 | 0.38 |

^a^General linear model for continuous variables, adjusted for age and sex when applicable. Examination of diet was also adjusted for method, season and energy intake. Chi^2^-test for categorical variables.

^b^P-value for ln-transformed values.

^c^A high score indicates a high level of leisure time physical activity.

^d^Among those reporting that they consumed alcohol during the year before baseline examinations.

**Table S3 - Correlation coefficients^a^ between energy adjusted intakes of components in a diet risk score for type 2 diabetes in 25,069 individuals from the Malmö Diet and Cancer cohort**

|  | Processed meat | Sugar-sweetened beverages | Whole grain | Coffee |
| --- | --- | --- | --- | --- |
| Processed meat | 1.00 | 0.04 (0.06, 0.03 )^b^ | -0.05 (-0.05, -0.03) | 0.08 (0.08, 0.09) |
| Sugar-sweetened  beverages |  | 1.00 | -0.04 (-0.06, -0.03) | -0.02 (-0.02, -0.04) |
| Whole grain |  |  | 1.00 | -0.02 (-0.02,-0.03) |
| Coffee |  |  |  | 1.00 |

^a^P<0.001 for all correlations.

^b^Correlation coefficients for women and men respectively.

**Table S4.** Hazard ratios of incident type 2 diabetes according to combinations of a weighted genetic risk score and a diet risk score based on intakes of processed meat, sugar-sweetened beverages (SSB), whole grain and coffee in 15,380 women and 9689 men from the Malmö Diet and Cancer study

|  | All | | | | Women | | | | Men | | | |
| --- | --- | --- | --- | --- | --- | --- | --- | --- | --- | --- | --- | --- |
| Diet risk score level/  diet tertile | Tertile of genetic risk score | | | P  value for trend^a^ | Tertile of genetic risk score | | | P value for trend^a^ | Tertile of genetic risk score | | | P  value for trend^a^ |
|  | 1 | 2 | 3 |  | 1 | 2 | 3 |  | 1 | 2 | 3 |  |
| Diet risk score |  |  |  |  |  |  |  |  |  |  |  |  |
| Low | 1.00^b,c^ | 1.39  1.14,1.70 | 1.80  1.49,2.18 | 4×10^-8^ | 1.00 | 1.49  1.13,1.98 | 1.86  1.42,2.43 | 6×10^-5^ | 1.00 | 1.30  0.98,0.73 | 1.73  1.32,2.28 | 2×10^-4^ |
| Medium | 1.23  1.03,1.46 | 1.62  1.37,1.92 | 2.15  1.82,2.53 | 2×10^-21^ | 1.48  1.15,1.88 | 1.86  1.46,2.36 | 2.47  1.96,3.13 | 4×10^-11^ | 1.03  0.80,1.30 | 1.43  1.12,1.81 | 1.90  1.48,2.36 | 8×10^-12^ |
| High | 1.43  1.16,1.76 | 1.94  1.59,2.38 | 2.49  2.06,3.01 | 1×10^-8^ | 1.65  1.22,2.22 | 2.15  1.61,2.87 | 2.57  1.95,3.38 | 0.002 | 1.24  0.92,1.66 | 1.78  1.34,2.34 | 2.38  1.83,3.10 | 2×10^-6^ |
| P_trend_^c^ | 0.001 | 3×10^-4^ | 8×10^-5^ |  | 0.001 | 0.006 | 0.005 |  | 0.20 | 0.01 | 0.005 |  |
| P_interaction_^d^ |  |  |  | 0.83  (0.94) |  |  |  | 0.40  (0.67) |  |  |  | 0.60  (0.73) |

^a^ Age stratified model, adjusted for sex when applicable

^b^ Reference in joint effect model

^c^ Age stratified model, adjusted for sex, diet method version, season, total energy intake, BMI, leisure time physical activity, alcohol intake, smoking and education

^d^P for interaction treating tertiles as continuous variables, and in brackets P for interaction between continuous variables of the GRS and the DRS.

**Table S5.** HR of incident type 2 diabetes according to combinations of a weighted genetic risk score and components of a diet risk score in 15,380 women and 9689 men from the Malmö Diet and Cancer study

|  | All | | | | Women | | | | Men | | | |
| --- | --- | --- | --- | --- | --- | --- | --- | --- | --- | --- | --- | --- |
| Diet tertile | Tertile of genetic risk score | | | P  value for trend^a^ | Tertile of genetic risk score | | | P value for trend^a^ | Tertile of genetic risk score | | | P  value for trend^a^ |
|  | 1 | 2 | 3 |  | 1 | 2 | 3 |  | 1 | 2 | 3 |  |
| Processed meat |  |  |  |  |  |  |  |  |  |  |  |  |
| 1 | 1.00^b,c^ | 1.31  1.12,1.54 | 1.73  1.48,2.01 | 1×10^-11^ | 1.00 | 1.37  1.10,1.70 | 1.78  1.45,2.20 | 1×10^-7^ | 1.00 | 1.27  1.00,1.60 | 1.70  1.36,2.13 | 2×10^-5^ |
| 2 | 1.02  0.87,1.21 | 1.31  1.12,1.53 | 1.80  1.55,2.09 | 2×10^-11^ | 1.19  0.95,1.49 | 1.37  1.10,1.71 | 1.86  1.51,2.29 | 6×10^-5^ | 0.90  0.71,1.14 | 1.27  1.01,1.60 | 1.81  1.45,2.25 | 3×10^-8^ |
| 3 | 1.08  0.92,1.27 | 1.52  1.31,1.78 | 1.92  1.66,2.23 | 1×10^-14^ | 1.14  0.91,1.43 | 1.61  1.30,1.99 | 1.97  1.60,2.42 | 5×10^-7^ | 1.04  0.83,1.31 | 1.48  1.18,1.82 | 1.90  1.53,2.35 | 1×10^-8^ |
| P_trend_^c^ | 0.61 | 0.04 | 0.03 |  | 0.47 | 0.11 | 0.18 |  | 0.76 | 0.18 | 0.12 |  |
| P_interaction_^d^ |  |  |  | 0.84  (0.49) |  |  |  | 0.79  (0.70) |  |  |  | 0.80  (0.64) |
| SSB |  |  |  |  |  |  |  |  |  |  |  |  |
| 1 | 1.00 ^b,c^ | 1.38  1.21,1.58 | 1.85  1.63,2.09 | 1×10^-17^ | 1.00 | 1.30  1.08,1.56 | 1.73  1.45,2.06 | 8×10^-8^ | 1.00 | 1.48  1.22,1.79 | 1.95  1.62,2.34 | 2×10^-11^ |
| 2 | 1.11 0.94,1.30 | 1.39  1.19,1.61 | 1.75  1.52,2.02 | 5×10^-8^ | 1.16  0.92,1.47 | 1.39  1.11,1.73 | 1.69  1.38,2.08 | 0.001 | 1.02  0.82,1.28 | 1.35  1.09,1.67 | 1.79  1.47,2.19 | 2×10^-5^ |
| 3 | 1.15  0.99,1.34 | 1.56  1.35,1.80 | 2.07  1.80,2.37 | 2×10^-12^ | 1.08  0.88,1.33 | 1.51  1.24,1.84 | 1.95  1.62,2.35 | 4×10^-8^ | 1.23  0.97,1.54 | 1.57  1.27,1.95 | 2.19  1.78,2.68 | 1×10^-5^ |
| P_trend_^c^ | 0.05 | 0.07 | 0.16 |  | 0.25 | 0.10 | 0.29 |  | 0.18 | 0.48 | 0.42 |  |
| P_interaction_^d^ |  |  |  | 0.69  (0.92) |  |  |  | 0.98  (0.84) |  |  |  | 0.54  (0.90) |
| Wholegrain |  |  |  |  |  |  |  |  |  |  |  |  |
| 1 | 1.00 ^b,c^ | 1.45  1.25,1.68 | 1.80  1.57,2.07 | 9×10^-16^ | 1.00 | 1.34  1.08,1.66 | 1.50  1.23,1.84 | 2×10^-5^ | 1.00 | 1.57  1.29,1.92 | 2.18  1.80,2.64 | 2×10^-12^ |
| 2 | 1.06  0.92,1.24 | 1.22  1.05,1.42 | 1.73  1.50,2.00 | 6×10^-9^ | 0.99  0.79,1.22 | 1.10  0.89,1.36 | 1.61  1.31,1.96 | 1×10^-5^ | 1.15  0.92,1.43 | 1.36  1.09,1.68 | 1.86  1.51,2.29 | 2×10^-4^ |
| 3 | 0.88  0.75,1.04 | 1.28  1.10,1.49 | 1.63  1.41,1.89 | 9×10^-13^ | 0.78  0.62,0.98 | 1.19  0.97,1.48 | 1.54  1.26,1.88 | 2×10^-8^ | 1.00  0.79,1.26 | 1.37  1.10,1.69 | 1.69  1.37,2.07 | 8×10^-6^ |
| P_trend_^c^ | 0.11 | 0.06 | 0.14 |  | 0.03 | 0.32 | 0.80 |  | 0.64 | 0.09 | 0.003 |  |
| P_interaction_^d^_._ |  |  |  | 0.81  (0.88) |  |  |  | 0.07  (0.27) |  |  |  | 0.07  (0.35) |
| Coffee |  |  |  |  |  |  |  |  |  |  |  |  |
| 1 | 1.00 ^b,c^ | 1.40  1.21,1.62 | 1.82  1.59,2.09 | 1×10^-14^ | 1.00 | 1.45  1.19,1.77 | 1.81  1.50,2.18 | 1×10^-7^ | 1.00 | 1.35  1.09,1.67 | 1.84  1.51,2.25 | 2×10^-8^ |
| 2 | 0.87  0.75,1.02 | 1.21  1.04,1.40 | 1.60  1.39,1.84 | 2×10^-12^ | 0.84  0.68,1.04 | 1.05  0.85,1.28 | 1.41  1.16,1.71 | 7×10^-6^ | 0.90  0.72,1.14 | 1.40  1.13,1.73 | 1.83  1.49,2.24 | 4×10^-8^ |
| 3 | 0.81  0.69,0.95 | 1.02  0.88,1.19 | 1.31  1.14,1.52 | 1×10^-10^ | 0.69  0.55,0.86 | 0.87  0.71,1.08 | 1.10  0.90,1.34 | 3×10^-6^ | 0.94  0.75,1.18 | 1.18  0.95,1.46 | 1.58  1.28,1.95 | 8×10^-6^ |
| P_trend_^c^ | 0.01 | 2×10^-5^ | 1×10^-6^ |  | 0.001 | 8×10^-7^ | 2×10^-7^ |  | 0.38 | 0.25 | 0.15 |  |
| P_interaction_^d^ |  |  |  | 0.29  (0.60) |  |  |  | 0.44  (0.86) |  |  |  | 0.56  (0.36) |

^a^ Age stratified model, adjusted for sex when applicable

^b^ Reference in joint effect model

^c^ Age stratified model, adjusted for sex, diet method version, season, total energy intake, BMI, leisure time physical activity, alcohol intake, smoking and education

^d^P for interaction treating tertiles as continuous variables, and in brackets P-for interaction between continuous variables of the GRS and the DRS.

**Table S6.** HR^a^ of incident type 2 diabetes according to extended dietary risk scores (DRS) for type 2 diabetes and the added dietary components in 15,380 women and 9689 men from the Malmö Diet and Cancer cohort

|  | All | | | | Women | | | | Men | | | |
| --- | --- | --- | --- | --- | --- | --- | --- | --- | --- | --- | --- | --- |
|  | Low  (0-3) | Medium  (4-6) | High  (7-10) | P-trend | Low  (0-3) | Medium  (4-6) | High  (7-10) | P-trend | Low  (0-3) | Medium  (4-6) | High  (7-10) | P-trend |
| DRS^b^ with fruit and vegetables | 1.00 | 1.23  1.13, 1.33 | 1.37  1.24, 1.51 | 9×10^-10^ | 1.00 | 1.33  1.18, 1.48 | 1.45  1.26, 1.67 | 4×10^-8^ | 1.00 | 1.15  1.01, 1.30 | 1.29  1.11, 1.49 | 0.001 |
| DRS^b^ with fermented dairy | 1.00 | 1.21  1.11, 1.32 | 1.36  1.23, 1.50 | 2×10^-9^ | 1.00 | 1.32  1.18, 1.48 | 1.51  1.31, 1.73 | 4×10^-9^ | 1.00 | 1.10  0.98, 1.26 | 1.22  1.06, 1.41 | 0.005 |
| DRS^b^ with  high-fat fish | 1.00 | 1.18  1.09, 1.29 | 1.36  1.24, 1.51 | 1×10^-9^ | 1.00 | 1.30  1.15,1.46 | 1.54  1.34,1.77 | 4×10^-9^ | 1.00 | 1.07  0.94,1.20 | 1.21  1.04,1.40 | 0.01 |
|  | Low  (0-4) | Medium  (5-9) | High  (10-14) |  | Low  (0-4) | Medium  (5-9) | High  (10-14) |  | Low  (0-4) | Medium  (5-9) | High  (10-14) |  |
| DRS^b^ with fruits, vegetables, fermented dairy, high-fat fish | 1.00 | 1.18  1.08, 1.30 | 1.39  1.23, 1.56 | 8×10^-8^ | 1.00 | 1.21  1.07,1.37 | 1.45  1.23,1.71 | 9×10^-6^ | 1.00 | 1.17  1.00,1.36 | 1.30  1.14,1.63 | 0.001 |
|  | Tertile | | |  | Tertile | | |  | Tertile | | |  |
|  | 1 | 2 | 3 |  | 1 | 2 | 3 |  | 1 | 2 | 3 |  |
| Fruit and vegetable intake | 1.00 | 0.95  0.87, 1.03 | 0.95  0.87, 1.04 | 0.24 | 1.00 | 0.94  0.84, 1.06 | 0.95  0.84, 1.07 | 0.42 | 1.00 | 0.95  0.85, 1.06 | 0.93  0.82, 1.05 | 0.24 |
| Fermented  dairy intake | 1.00 | 0.96  0.89, 1.04 | 0.92  0.84, 1.00 | 0.05 | 1.00 | 0.96  0.86, 1.08 | 0.84  0.74, 0.94 | 0.002 | 1.00 | 0.96  0.86, 1.08 | 1.00  0.89, 1.14 | 0.98 |
| High fat fish intake | 1.00 | 1.00  0.93-1.09 | 0.96  0.87-1.04 | 0.37 | 1.00 | 0.99  0.88,1.11 | 0.99  0.88,1.10 | 0.82 | 1.00 | 1.00  0.90,1.13 | 0.92  0.82,1.05 | 0.24 |

^a^Age stratified model, adjusted for sex, diet method version, season, total energy intake, BMI, leisure time physical activity, alcohol intake, smoking and education

^b^The original DRS included intakes of processed meat, sugar-sweetened beverages, whole grain and coffee.

**Table S7.** HR of incident type 2 diabetes according to tertiles of a genetic risk score and alternative dietary risk scores (DRS) including additional diet components in 15,380 women and 9689 men from the Malmö Diet and Cancer cohort

| DRS^b^ | All | | | | Women | | | | Men | | | |
| --- | --- | --- | --- | --- | --- | --- | --- | --- | --- | --- | --- | --- |
|  | Tertile of genetic risk score | | | P_trend_^a^ | Tertile of genetic risk score | | | P_trend_^a^ | Tertile of genetic risk score | | | P_trend_^a^ |
|  | 1 | 2 | 3 |  | 1 | 2 | 3 |  | 1 | 2 | 3 |  |
| DRS with fruit and vegetables |  |  |  |  |  |  |  |  |  |  |  |  |
| Low | 1.00^c,d^ | 1.28  1.06,1.54 | 1.68  1.40,2.00 | 2×10^-7^ | 1.00 | 1.29  1.01,1.65 | 1.75  1.39,2.22 | 2×10^-5^ | 1.00 | 1.22  0.92,1.61 | 1.56  1.19,2.06 | 0.002 |
| Medium | 1.17  0.99,1.38 | 1.55  1.32,1.82 | 2.12  1.82,2.47 | 4×10^-23^ | 1.35  1.08,1.68 | 1.63  1.32,2.02 | 2.36  1.92,2.91 | 2×10^-12^ | 1.00  0.79,1.28 | 1.45  1.15,1.84 | 1.90  1.51,2.40 | 3×10^-12^ |
| High | 1.32  1.09,1.59 | 1.89  1.58,2.26 | 2.19  1.84,2.61 | 2×10^-7^ | 1.46  1.12,1.92 | 2.34  1.82,3.00 | 2.11  1.64,2.70 | 0.02 | 1.17  0.89,1.54 | 1.55  1.18,2.00 | 2.20  1.71,2.82 | 3×10^-6^ |
| P_trend_^d^ | 0.009 | 9×10^-6^ | 0.001 |  | 0.005 | 1×10^-6^ | 0.04 |  | 0.37 | 0.11 | 0.003 |  |
| P_interaction_ |  |  |  | 0.50 |  |  |  | 0.19 |  |  |  | 0.26 |
| DRS with fermented dairy |  |  |  |  |  |  |  |  |  |  |  |  |
| Low | 1.00^c,d^ | 1.34  1.11,1.61 | 1.74  1.45,2.09 | 3×10^-8^ | 1.00 | 1.38  1.07,1.79 | 1.88  1.47,2.41 | 3×10^-6^ | 1.00 | 1.24  0.94,1.63 | 1.58  1.21,2.06 | 0.002 |
| Medium | 1.22  1.04,1.45 | 1.57  1.34,1.85 | 2.16  1.84,2.53 | 4×10^-21^ | 1.46  1.16,1.84 | 1.78  1.42,2.24 | 2.44  1.96,3.04 | 8×10^-11^ | 1.01  0.79,1.28 | 1.36  1.08,1.71 | 1.91  1.52,2.40 | 9×10^-12^ |
| High | 1.31  1.08,1.59 | 1.96  1.63,2.35 | 2.27  1.91,2.71 | 7×10^-9^ | 1.60  1.22,2.11 | 2.36  1.82,3.06 | 2.52  1.95,3.24 | 0.002 | 1.07  0.81,1.40 | 1.60  1.23,2.06 | 2.00  1.56,2.56 | 8×10^-7^ |
| P_trend_^d^ | 0.008 | 3×10^-5^ | 4×10^-4^ |  | 0.001 | 2×10^-5^ | 0.004 |  | 0.77 | 0.07 | 0.03 |  |
| P_interaction._ |  |  |  | 0.92 |  |  |  | 0.27 |  |  |  | 0.42 |
| DRS with  high fat fish |  |  |  |  |  |  |  |  |  |  |  |  |
| Low | 1.00^c,d^ | 1.36  1.13,1.64 | 1.67  1.40,2.00 | 4×10^-7^ | 1.00^2^ | 1.26  0.97,1.63 | 1.59  1.24,2.03 | 0.001 | 1.00^2^ | 1.48  1.13,1.93 | 1.77  1.36,2.30 | 1×10^-4^ |
| Medium | 1.19  1.01,1.41 | 1.49  1.27,1.75 | 2.08  1.78,2.44 | 5×10^-22^ | 1.27  1.01,1.60 | 1.52  1.21,1.90 | 2.21  1.78,2.74 | 1×10^-12^ | 1.11  0.88,1.41 | 1.44  1.14,1.81 | 1.98  1.58,2.48 | 6×10^-11^ |
| High | 1.26  1.04,1.53 | 1.98  1.64,2.37 | 2.33  1.95,2.78 | 3×10^-9^ | 1.38  1.05,1.81 | 2.30  1.79,2.96 | 2.33  1.81,3.00 | 4×10^-4^ | 1.16  0.87,1.53 | 1.68  1.28,2.20 | 2.32  1.80,2.98 | 2×10^-6^ |
| P_trend_^d^ | 0.02 | 9×10^-6^ | 6×10^-5^ |  | 0.02 | 6×10^-7^ | 3×10^-4^ |  | 0.43 | 0.24 | 0.03 |  |
| P_interaction_ |  |  |  | 0.44 |  |  |  | 0.84 |  |  |  | 0.35 |
| DRS with fruits and vegetables, fermented dairy, high-fat fish |  |  |  |  |  |  |  |  |  |  |  |  |
| Low | 1.00^c,d^ | 1.42  1.14,1.78 | 1.74  1.40,2.17 | 5×10^-6^ | 1.00^2^ | 1.39  1.04,1.87 | 1.94  1.47,2.57 | 8×10^-6^ | 1.00^2^ | 1.48  1.04,2.10 | 1.41  0.97,2.04 | 0.11 |
| Medium | 1.22  1.01,1.47 | 1.57  1.30,1.90 | 2.16  1.80,2.60 | 4×10^-25^ | 1.34  1.04,1.72 | 1.68  1.31,2.15 | 2.26  1.78,2.88 | 7×10^-12^ | 1.09  0.82,1.46 | 1.45  1.09,1.94 | 2.05  1.54,2.72 | 9×10^-15^ |
| High | 1.39  1.10,1.75 | 2.05  1.64,2.56 | 2.36  1.91,2.93 | 7×10^-7^ | 1.63  1.18,2.26 | 2.41  1.71,3.26 | 2.30  1.77,3.12 | 0.02 | 1.20  0.85,1.69 | 1.78  1.29,2.47 | 2.36  1.72,3.24 | 4×10^-6^ |
| P_trend_^d^ | 0.009 | 0.002 | 0.002 |  | 0.002 | 0.0004 | 0.17 |  | 0.49 | 0.09 | 0.002 |  |
| P_interaction_ |  |  |  | 0.81 |  |  |  | 0.10 |  |  |  | 0.20 |

^a^ Age stratified model, adjusted for sex when applicable

^b^The original DRS included intakes of processed meat, sugar-sweetened beverages, whole grain and coffee

^c^ Reference in joint effect model

^d^ Age stratified model, adjusted for sex, diet method version, season, total energy intake, BMI, leisure time physical activity, alcohol intake, smoking and education

**Table S8.** HR of incident type 2 diabetes (T2D) according to tertiles of a genetic risk score and intakes of the additional components in the alternative dietary risk scores in 15,380 women and 9689 men from the Malmö Diet and Cancer study

|  | All | | | | Women | | | | Men | | | |
| --- | --- | --- | --- | --- | --- | --- | --- | --- | --- | --- | --- | --- |
|  | Tertile of genetic risk score | | | P_trend_^a^ | Tertile of genetic risk score | | | P_trend_^a^ | Tertile of genetic risk score | | | P_trend_^a^ |
| Tertile of dietary intakes | 1 | 2 | 3 |  | 1 | 2 | 3 |  | 1 | 2 | 3 |  |
| Fruits and vegetables |  |  |  |  |  |  |  |  |  |  |  |  |
| 1 | 1.00^b,c^ | 1.29  1.12,1.48 | 1.60  1.40,1.84 | 3×10^-9^ | 1.00 | 1.43  1.14,1.80 | 1.46  1.16,1.83 | 0.003 | 1.00 | 1.20  1.00,1.44 | 1.72  1.45,2.05 | 2×10^-7^ |
| 2 | 0.86  0.73,1.00 | 1.25  1.08,1.45 | 1.61  1.40,1.85 | 3×10^-15^ | 0.95  0.75,1.19 | 1.20  0.96,1.50 | 1.57  1.26,1.95 | 9×10^-7^ | 0.75  0.60,0.94 | 1.31  1.08,1.59 | 1.66  1.37,2.01 | 9×10^-10^ |
| 3 | 0.90  0.77,1.06 | 1.16  0.99,1.34 | 1.64  1.43,1.90 | 5×10^-14^ | 0.91  0.72,1.15 | 1.13  0.90,1.41 | 1.70  1.38,2.10 | 9×10^-10^ | 0.90  0.71,1.13 | 1.19  0.96,1.47 | 1.52  1.24,1.87 | 2×10^-5^ |
| P_trend_^c^ | 0.26 | 0.12 | 0.67 |  | 0.43 | 0.03 | 0.13 |  | 0.39 | 0.99 | 0.29 |  |
| P_interact._ |  |  |  | 0.16 |  |  |  | 0.04 |  |  |  | 0.91 |
| Fermented dairy |  |  |  |  |  |  |  |  |  |  |  |  |
| 1 | 1.00^b,c^ | 1.29  1.12,1.48 | 1.67  1.46,1.91 | 3×10^-12^ | 1.00 | 1.22  1.00,1.51 | 1.48  1.21,1.80 | 0.001 | 1.00 | 1.36  1.12,1.64 | 1.83  1.53,2.20 | 3×10^-10^ |
| 2 | 0.94  0.80,1.09 | 1.26  1.09,1.45 | 1.63  1.42,1.87 | 2×10^-11^ | 0.86  0.69,1.07 | 1.22  1.00,1.50 | 1.47  1.20,1.78 | 8×10^-7^ | 1.01  0.81,1.24 | 1.25  1.02,1.54 | 1.81  1.49,2.20 | 7×10^-6^ |
| 3 | 0.85  0.72,0.99 | 1.18  1.02,1.37 | 1.59  1.38,1.83 | 5×10^-15^ | 0.74  0.59,0.92 | 0.93  0.76,1.15 | 1.41  1.16,1.71 | 5×10^-10^ | 0.96  0.75,1.22 | 1.47  1.19,1.81 | 1.78  1.44,2.19 | 2×10^-6^ |
| P_trend_^c^ | 0.048 | 0.44 | 0.29 |  | 0.007 | 0.02 | 0.56 |  | 1.00 | 0.26 | 0.42 |  |
| P_interact._ |  |  |  | 0.26 |  |  |  | 0.07 |  |  |  | 0.94 |
| High-fat fish |  |  |  |  |  |  |  |  |  |  |  |  |
| 1 | 1.00^b,c^ | 1.42  1.22,1.65 | 1.90  1.64,2.19 | 6×10^-15^ | 1.00 | 1.59  1.29,1.95 | 1.90  1.55,2.33 | 2×10^-8^ | 1.00 | 1.23  0.99,1.52 | 1.90  1.55,2.33 | 8×10^-8^ |
| 2 | 1.12  0.96,1.31 | 1.37  1.17,1.59 | 1.82  1.58,2.10 | 9×10^-10^ | 1.22  0.98,1.53 | 1.35  1.08,1.70 | 1.82  1.48,2.24 | 2×10^-4^ | 1.00  0.80,1.24 | 1.34  1.08,1.66 | 1.78  1.46,2.18 | 1×10^-6^ |
| 3 | 1.00  0.85,1.18 | 1.38  1.18,1.60 | 1.74  1.51,2.02 | 1×10^-13^ | 1.12  0.90,1.40 | 1.38  1.11,1.71 | 1.89  1.54,2.31 | 3×10^-7^ | 0.88  0.69,1.11 | 1.35  1.09,1.67 | 1.57  1.27,1.94 | 6×10^-8^ |
| P_trend_^c^ | 0.93 | 0.34 | 0.42 |  | 0.37 | 0.10 | 0.98 |  | 0.43 | 0.80 | 0.24 |  |
| P_interact._ |  |  |  | 0.40 |  |  |  | 0.54 |  |  |  | 0.53 |

^a^ Age stratified model, adjusted for sex when applicable

^b^ Reference in joint effect model

^c^ Age stratified model, adjusted for sex, diet method version, season, total energy intake, BMI, leisure time physical activity, alcohol intake, smoking and education

**Table S9. Additional single nucleotide polymorphisms included in the extended GRS and reported to associate with type 2 diabetes by Fuchsberger et al 2016.**

| SNP | Chromosome: position | Function* | Minor Allele Frequency | Minor Allele | Risk allele |
| --- | --- | --- | --- | --- | --- |
| 1. PEPD rs8182584 | 19:33418804 | intron | 0.10 | C | C |
| 1. C2CD4A/B rs4502156 | 15:62090956 | intergenic | 0.45 | C | T |
| 1. CCND2 rs11063069 | 12:4265207 | intron | 0.20 | G | G |
| 1. DUSP8 rs2334499 | 11:1675619 | intergenic | 0.43 | T | T |
| 1. CDC123 rs11257655 | 10:12265895 | intergenic | 0.22 | T | T |
| 1. GRK5 rs10886471 | 10:119389891 | intron | 0.47 | T | C |
| 1. GLIS3 rs10758593 | 9:4292083 | intron | 0.40 | A | A |
| 1. RASGRP1 rs7403531 | 5:38530704 | intron | 0.08 | G | A |
| 1. GCK rs6975024 | 7:44192287 | intergenic | 0.16 | C | C |
| 1. SSR1/REB1 rs1334577 | 6:7211518 | intron | 0.22 | A | A |
| 1. ARL15 rs4865796 | 5:53976834 | intron | 0.31 | G | A |
| 1. MAEA rs10025665 | 4:1318479 | intron | 0.01 | G | A |
| 1. TMEM154 rs6813195 | 4:152599323 | intergenic | 0.29 | T | C |
| 1. ST6GALI rs7648806 | 3:186898516 | intergenic | 0.13 | T | T |
| 1. UBE2E2 rs1496653 | 3:23413299 | intron | 0.22 | G | A |
| 1. PSMD6 rs7621907 | 3:64106541 | intron | 0.17 | A | G |
| 1. SSR rs391300 | 17:2312964 | intron | 0.39 | T | T |
| 1. FAF1 rs1278516 | 1:50434349 | intergenic | 0.11 | A | G |
| 1. LAMA rs7240767 | 18:7070643 | intron | 0.37 | C | C |
| 1. SPRY2 rs1359790 | 13:80143021 | intron | 0.26 | A | G |

*According to the human reference genome *GRCh38*, released from the [Genome Reference Consortium](https://en.wikipedia.org/wiki/Genome_Reference_Consortium) 2013
