# Supplementary material for: Dietary and genetic risk scores and incidence of type 2 diabetes
Source: Genes Nutr. 2018 May 16;13:13. doi: 10.1186/s12263-018-0599-1 (PMC5956794; doi:10.1186/s12263-018-0599-1)
Supplement: Supplementary file 2 — Figure S1. In a subsample (n = 4193), with genetic data on 68 T2D SNPs (20 additional SNPs), the findings were similar to those in the whole study sample. No statistical interaction was observed between the extended genetic risk score and the dietary risk scores (P = 0.34). Individuals with both high genetic susceptibility and unfavourable dietary habits had more than twice as high risk (HR: 3.82; 95% CI: 2.18, 6.71) of developing T2D compared to those with low genetic susceptibility and favourable dietary habits (reference HR = 1.00). (DOCX 37 kb) [file 12263_2018_599_MOESM2_ESM.docx]

Low GRS68 Medium GRS68 High GRS68

*P for interaction=0.34*

**Figure legend.**

In a subsample (n=4,193), with genetic data on 68 T2D SNPs (20 additional SNPs), the findings were similar to those in the whole study sample. No statistical interaction was observed between the extended genetic risk score and the dietary risk scores (P=0.34). Individuals with both high genetic susceptibility and unfavourable dietary habits had more than twice as high risk (HR: 3.82; 95% CI: 2.18, 6.71) of developing T2D compared to those with low genetic susceptibility and favourable dietary habits (reference HR=1.00).
